# The energetics and thermoregulation of water collecting honeybees

**DOI:** 10.1007/s00359-018-1278-9

**Published:** 2018-08-06

**Authors:** Helmut Kovac, Helmut Käfer, Anton Stabentheiner

**Affiliations:** Institute of Biology, University of Graz, Universitätsplatz 2, 8010 Graz, Austria

**Keywords:** Honeybee, Energetics, Thermoregulation, Water, Collecting

## Abstract

Honeybees need water for different purposes, to maintain the osmotic homeostasis in adults as well as to dilute stored honey and prepare liquid food for the brood. Water is also used for cooling of the hive. Foraging in endothermic insects is energy-intensive and the question arises how much energy bees invest in a resource without any metabolically usable energy. We investigated the energy demand of water collecting bees under natural conditions. The thermoregulation and energetic effort was measured simultaneously in a broad range of experimental ambient temperatures (*T*_a_ = 12–40 °C). The thorax temperature as well as the energetic turnover showed a great variability. The mean *T*_thorax_ was ranging from ~ 35.7 °C at 12 °C to nearly 42.5 °C at 40 °C. The energy turnover calculated from CO_2_-release was highest at a *T*_a_ of 20 °C with about 60 mW and lowest at 40 °C with about 22 mW per bee. The total costs during collection decreased from 10.4 J at 12 °C to 0.5 J at 40 °C. The energetic effort of the water collectors was comparable with that of 0.5 M sucrose foraging bees. Our investigation strongly supports the hypothesis that the bees’ motivational status determines the energetic performance during foraging.

**Electronic supplementary material:**

The online version of this article (10.1007/s00359-018-1278-9) contains supplementary material, which is available to authorized users.

## Introduction

Besides pollen and nectar, water is an essential resource for the honeybee colony. Water is needed to maintain the osmotic homeostasis in adult bees, but also to dilute stored honey and prepare liquid food for the brood. On hot days water is used to cool the hive (for a review see e.g. Seeley 1985; Heinrich 1993). By means of cooling and endothermic heat production, the temperature in the brood nest area is regulated within a narrow range of about 32–35 °C (e.g. Hess 1926; Himmer 1927; Büdel 1955; Simpson 1961; Stabentheiner et al. 2010; Sachs and Tautz 2017). If the temperature in the brood nest exceeds a certain threshold, foragers start to collect water. Back in the hive, they unload the water to hive bees, which disperse it on the combs for evaporative cooling (Lindauer 1952, 1954, 1955; Visscher et al. 1996). Lindauer (1954) and Kühnholz and Seeley (1997) demonstrated that the motivation of foragers to continue collecting water depends on the time it takes inside the hive to unload their crop content. The results of Kühnholz and Seeley (1997) strongly confirm the idea of Lindauer (1954) that the foragers’ decision to collect water is not based on direct measurements of their colony’s water supply and demand, but on an indirect indicator of the supply–demand ratio, namely the ease of unloading water. In a further study, Ostwald et al. (2016) clarified how a colony’s water collectors know when to spring into action. Aside the necessity of cooling the hive on warm days, honeybees need water urgently in late winter and early spring. At those times they forage for water at ambient temperatures below 12 °C (down to ~ 5 °C; Kovac et al. 2010; Chilcott and Seeley 2018), temperatures they usually avoid even if rich nectar sources are available (Kovac and Stabentheiner 2011).

Water collecting honeybees exhibit a high body temperature, even at low ambient temperatures (Schmaranzer 2000; Kovac et al. 2010). A high body temperature accomplished by endothermic activity means a high energetic expenditure. Such a high energetic investment has been observed in bees foraging at sources with a high energetic reward (sucrose), with a graduated energetic investment, depending on the energetic gain (Stabentheiner and Kovac 2014, 2016). It is a well-known fact that foraging strategies of social insects balance the energy expenditure of the individual foragers with the net energetic gain of the colony (e.g. Seeley 1986, 1994; Seeley et al. 1991; Varjú and Núñez 1991, 1993; Balderrama et al. 1992; Kovac et al. 2018). However, water has no usable energetic value. How could the (high) energetic investment be explained in this case? A possible explanation is the bees’ “motivation”. Motivation is an important parameter modulating the thermoregulatory behaviour and energetics in bees collecting materials (food, water, and resin) for their colony. The bees’ motivational status depends e.g. on the quality and quantity of the nectar, the demand in the hive for nectar, and the patch distance from the hive. Bees foraging on resources with a high reward rate (e.g. sucrose concentration and flow rate) exhibit a higher body temperature and metabolic performance than bees foraging on energetically poor sources (see e.g. Dyer and Seeley 1987; Stabentheiner and Schmaranzer 1987; Schmaranzer and Stabentheiner 1988; Stabentheiner and Hagmüller 1991; Balderrama et al. 1992; Moffatt and Núñez 1997; Moffatt 2000, 2001; Stabentheiner 1996, 2001; Sadler and Nieh 2011; Stabentheiner and Kovac 2014, 2016; Waddington and Holden 1979; Waddington 1990). The concept that the motivational status influences foraging energetics could also be applied to pollen or water collectors.

In this study, we investigated for the first time the energetic demand of water collecting honeybees to see how much energy they are willing to invest in a resource without any metabolically usable energy content. Therefore, we measured the energetic effort during the collecting stay at a water source for drinking water and preparing for departure. We tested the hypothesis that the energetic gain is not the only parameter determining the energetic investment in foraging bees, but rather the bees’ “motivation” is responsible for their energetic effort. We compared the energetic demand of water collecting bees with that of sucrose foraging bees (Stabentheiner and Kovac 2016). With our results we can convincingly demonstrate that motivation is an important parameter in foraging bees that modulates thermoregulatory behaviour and energetic performance.

## Materials and methods

### Location, experimental design and procedure

The experiments were conducted in spring and summer in two consecutive years (2009 and 2010) in an orchard in Gschwendt/Austria, housing 10 honeybee colonies (*Apis mellifera carnica*). Bees were lured and trained to collect water at an artificial water source. The water source was near a building (1 m) endowed with an external laboratory facility which was equipped with the measurement devices (respiration and temperature measurement). The bees collected spontaneously from the water source. Pure water without any added substances was offered in sufficient amount in a small plastic cup. Eight foraging bees were marked individually with colour dots at the abdomen, but we measured also some unmarked bees. We estimate that at least 15 different bees collected at the water source and were subsequently measured. One marked individual was trained to collect water at a balance (AB104, Mettler-Toledo) to evaluate the crop load. The bee was weighed before and after foraging to the nearest 0.1 mg.

**Fig. 1:**
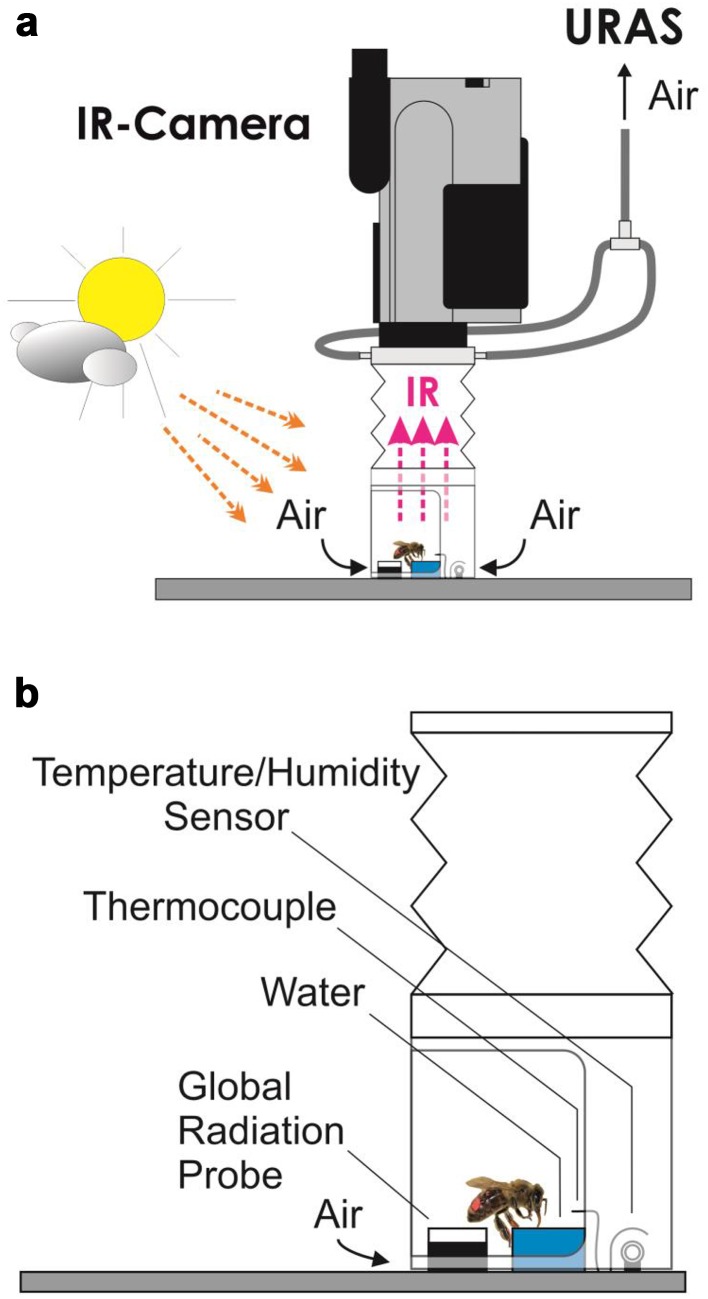
Experimental setup for the measurements carried out in 2010. **a** Schematic graph of the measurement chamber and the infrared camera. **b** Detail of the measurement chamber with placement of sensors

For body temperature and respiration measurements, bees collected water from a small plastic cup. For the respiration measurement, this cup was placed inside a small plastic cylinder connected to a gas analysing system. Immediately after landing, the cylindrical measurement chamber was put over the forager and opened manually after the bee had finished drinking. The duration of a foraging stay at the water source was defined as the time from landing at the water source, sucking water, preparation for take-off (often accompanied by grooming, with no imbibition of water), till leaving the measurement chamber. In the first series of experiments, body temperature and respiration were measured in different bees, but at the same time at identical water sources positioned close to each other (about 50 cm, for methodical details see also Stabentheiner et al. 2012). For the second experimental series, an improved measurement chamber was constructed, which allowed the simultaneous measurement of body temperature and respiration (Fig. 1). In these experiments, we used a bigger cylindrical chamber, where the upper and lower parts of the chamber were made of transparent acrylic glass. In the front of the lower part (diameter: 6 cm, height: 7 cm) a 6 × 6 cm window was cut out and covered by a transparent plastic film to allow as much as possible solar radiation to enter the measurement chamber. The larger chamber size also reduced heating in sunshine. The middle part of the chamber was a polyethylene foil bellows, which enabled opening the chamber by lifting the lower part to let the bee in and out. The upper part of the chamber was closed with the infrared camera lens.

### Environmental parameters: ambient temperature and solar radiation

The environmental conditions in the measurement chamber and outside were continuously monitored. Ambient air temperature, relative humidity and solar radiation were recorded in 1-second-intervals and stored using a data logger (Almemo 2890-9, Ahlborn; for details see Stabentheiner et al. 2012). The ambient air temperature in the chamber was measured about 1 cm beside the collecting bees by a Type K thermocouple and with a combined temperature / relative humidity sensor (Fig. 1b). The solar radiation was measured using a custom manufactured photoelectric miniature global radiation sensor (FLA613GS/Mini spezial, measurement range of 380–1100 nm; Ahlborn). The measurements were conducted in the shade and in the sun and sometimes under cloudy conditions. Results are divided into two categories according to the mean solar radiation during the collecting stay. The values below 370 W m^−2^ are defined as “shade” (mean = 55 ± 50 W m^−2^), the values above 370 W m^−2^ are defined as “sun” (mean = 635 ± 111 W m^−2^).

### Energy turnover

This study only examined the energetic effort of water collection from an artificial water source and not the costs of travel from and to the hive and the unloading process in the colony. The bees’ energy turnover was determined from their respiratory metabolism (CO_2_-production) which is commonly used as an indirect measure of an organism’s metabolic rate. Air was sucked off from the upper part of the measurement chamber (Fig. 1a). The CO_2_ content of the air was measured with a flow-through measurement setup in parallel mode using a differential infrared gas analyser (DIRGA; URAS 14, ABB), which was operating at a flow rate of about 700 ml/min. The digital data readout via the RS-232 interfaces of the DIRGA was done by Centrol 5 software (Harnisch, Austria). Depending on the experimental situation (duration of stay, influenced by ambient temperature and insolation), the rise and decline (washout) times of the CO_2_ signal resembled or even exceeded the visit duration. Thus, the insects’ energy turnover could not be measured by cutting out a section of the respiratory trace and simple averaging. Therefore, we integrated the bees’ total CO_2_ emission per stay (including 2 min of washout) and divided the integral by the duration of the stay inside the respiratory chamber. The loss of measurement gas during the opening of the chamber after the insects’ visits was compensated for by calibrations as described in Stabentheiner et al. (2012). Briefly, CO_2_ was injected into the measurement chamber via a syringe by a perfusor to achieve a stable measurement signal. Then the perfusor was turned off and the chamber was kept closed, or the perfusor was turned off and the chamber was opened for about 2 s (the period of the chamber opening when a bee left the chamber). During this period, the chamber was flushed with fresh air because the pump and mass flow controller were still active. In this way, we obtained two calibration curves of the CO_2_-amount in the system in dependence on the ‘turnover’ (concentration × flow) at the time when the perfusor was turned off. The difference between these two curves represented the CO_2_ loss caused by the chamber opening (Stabentheiner et al. 2012).

For the energetic calculation, we used a respiratory quotient of 1.0 (Stabentheiner and Kovac 2016) as honeybees use sugar as fuel for their flight activity. Therefore, the energy turnover (P) could be calculated directly from the CO_2_ production rate (VCO_2_) without the need to convert to O_2_ consumption: P [W] = VCO_2_ [lO_2_ s^−1^] × caloric equivalent [21 117 J lO_2_^−1^].

### Water collector’s body temperature

**Fig. 2:**
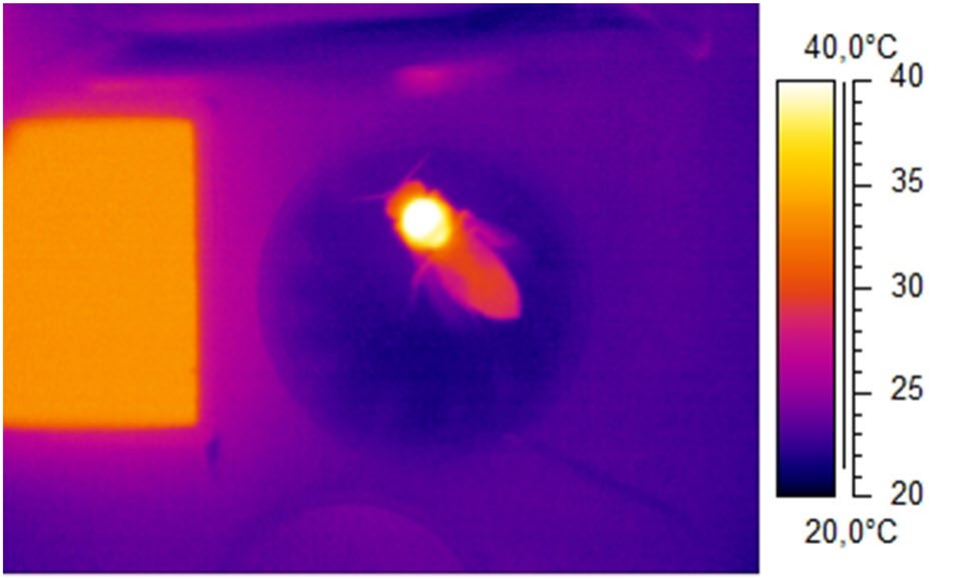
Thermogram of a water collecting honeybee. Body temperature: *T*_head_ = 33.3 °C, *T*_thorax_ = 39.4 °C, *T*_abdomen_ = 30.0 °C (*T*_ambient_ = 23.1 °C). Left-hand rectangle: proprietary infrared reference radiator

The upper part of the cylindrical measurement chamber was connected to the objective of an infrared camera (FLIR ThermaCam SC2000 NTS, Fig. 1a). This allowed the thermographic measurement of the bees’ body surface temperature and the observation of their behaviour during foraging. The infrared camera was calibrated against a proprietary Peltier-driven reference radiator placed close to the bees in the measurement chamber, visible within the infrared picture (Fig. 2; accuracy ≤ 0.4 °C; Stabentheiner et al. 2012). The body surface temperature was calibrated using the cuticular emissivity of honeybees (*ε* = 0.97; Stabentheiner and Schmaranzer 1987). The thermograms were stored digitally with 14 bit resolution at a rate of 5 Hz on a DOLCH FlexPac PC (Kontron) with the ThermaCAM Researcher software (FLIR). The thermographic measurements were evaluated with ThermaCAM Researcher Pro 2.10 software (FLIR) controlled by a proprietary MS Excel (Microsoft) VBA macro. This macro also extracted the stored environmental data automatically from the logger files at the time of thermographic measurement. The thermoregulatory behaviour was evaluated during the entire visit at the water source in a way that thermograms were taken every 3–5 s. From these thermograms the bees’ surface temperatures of head, thorax and abdomen were calculated.

### Data analysis

We measured the three parameters metabolic rate (CO_2_-release), body temperature and duration of collecting. The costs of collecting were calculated by means of the metabolic data and the duration of collecting. We analysed the measured and evaluated parameters (metabolic data, thoracic temperature, collecting time, costs of collecting) in dependence on ambient temperature and solar radiation. The metabolic data were evaluated in MS Excel (Microsoft Corporation) and Origin 2017 software (OriginLab). Curve fitting and statistics were done with Origin (OriginLab) and Statgraphics (Statgraphics Centurion XVI, StatPoint Technology Inc.) software. First, “General Linear Model (GLM)” statistics was performed to test the influence of the ambient temperature and solar radiation on the measured and calculated parameters. Furthermore, simple linear regressions and complex curve fittings (exponential and polynomial) in combination with an ANOVA were performed to represent and test the dependence of the parameters on ambient temperature. The average values for the evaluated parameters mentioned in the results derive from these fit curves. We compared the data of the water collectors (in shade) with sucrose foragers (in shade, see Stabentheiner and Kovac 2016). We refrained from comparison of foragers in the sun as in the two studies the range of solar radiation to which the bees were exposed to differed strongly. The constants of the fit curves and the statistical details are provided in Tables S1, S2 and S3. The experiments of the water collectors and the sucrose foragers (Stabentheiner and Kovac 2016) were conducted at the same location.

## Results

### Energetics and temperature

**Fig. 3:**
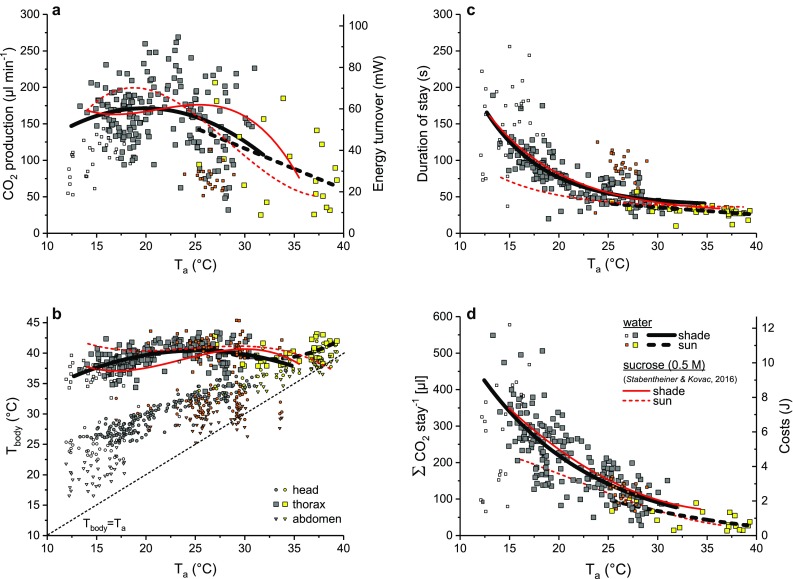
Energetics and thermoregulation of bees collecting water in the shade (grey and open symbols and black solid line) and in the sun (yellow and orange symbols and black broken line) in dependence on experimental ambient temperature (*T*_a_) near the bees in the measurement chamber. **a** Metabolic rate (CO_2_ release) and energy turnover, **b** body surface temperature, **c** duration of collecting stay, **d** costs (calculated from CO_2_-release and duration). Symbols represent mean values of the collecting stays. Red solid (shade) and broken (sun) lines represent data of sucrose collecting bees (0.5 M, from Stabentheiner and Kovac 2016). For constants of the fit curves and statistical details see Tables S1, S2 and S3

We evaluated 286 collecting visits of the bees at a broad range of experimental ambient temperature (*T*_a_ = 12–40 °C). In the first series in 2009 we measured 31 visits in the shade (< 370 W m^−2^) and 24 visits in the sun (> 370 W m^−2^). In the second series in 2010, we measured 191 visits in the shade and 40 visits in the sun. The curve fitting to the data was only performed for the second series (2010), as in the first series (2009) metabolism and body temperature were not measured in the same individuals. Variability of the metabolic rate data and the calculated energy turnover was very high (Fig. 3a). The general linear model (GLM) statistics revealed a strong dependence on radiation, but no dependence on ambient temperature (see Table S2). However, regression analysis showed a decrease with increasing ambient temperature (*p* < 0.05, ANOVA; see also Table S1) though this was not the case at low–medium temperatures (below ~ 25 °C). Metabolic rate was on average highest at an ambient temperature of 20 °C (shade) with about 60 mW and lowest at 40 °C (sun) with about 22 mW per bee (values derived from fit curves). The absolute lowest value was 9 mW and the highest observed value was 94 mW per bee (Fig. 3a).

The variability of the thorax temperature was also very high (Fig. 3b), but the GLM statistics showed a strong dependence on ambient temperature (Table S2). The average thorax temperature increased with ambient temperature (*p* < 0.01, ANOVA, Table S1). Mean values per stay ranged from about 35.7 to 39.8 °C at an ambient temperature of 12 and 30 °C, respectively (shade), and to nearly 42.5 °C at 40 °C (sun, Fig. 3b). The temperature of the head and the abdomen was always lower and depended more on the ambient temperature. In some experiments, the abdominal temperature was lower than the ambient air temperature. This could happen when the air outside the measurement chamber was cooler than inside and the abdomen was close to (outside) ambient temperature. The fit curves and detailed statistics for the metabolism and the thorax temperature are provided in Tables S1, S2 and S3.

### Duration and energetic costs of loading

The duration of the collecting stays depended strongly on ambient temperature, but not on radiation (GLM statistics, Table S2). It decreased in an exponential decay with *T*_a_ (*p* < 0.0001, ANOVA). In the shade, at an ambient temperature of 12 °C, a foraging stay lasted on average 181 s but only 45 s at 30 °C. At 40 °C, in the sun, the duration of a foraging stay was reduced to 25 s (Fig. 3c). It was noticeable that the bees in the first series needed more time to fill their crop than in the second series (especially in the sun).

**Fig. 4:**
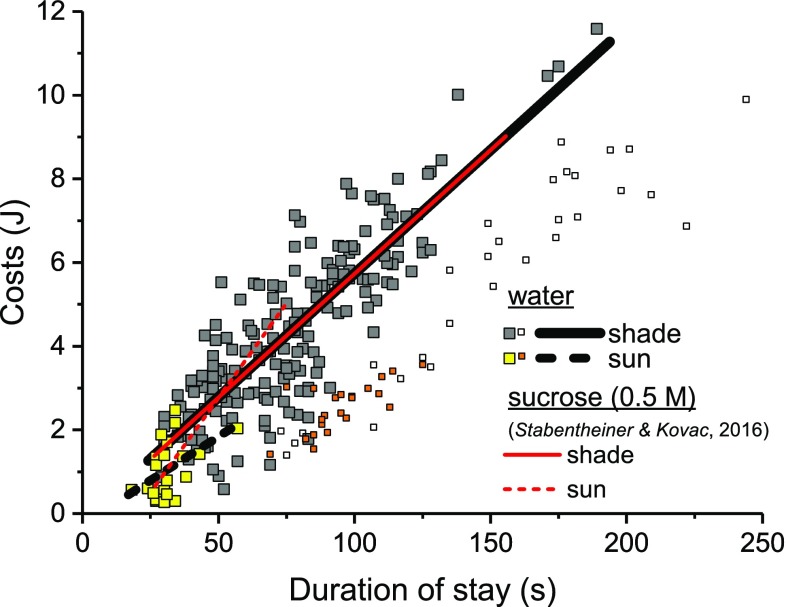
Costs of bees collecting water in the shade (grey and open symbols and black solid line) and in the sun (yellow and orange symbols and black broken line) in dependence on the duration of the collecting stays. Symbols represent mean values of the collecting stays. Red solid (shade) and broken (sun) lines represents data of sucrose foraging bees (0.5 M, from Stabentheiner and Kovac 2016). For constants of the fit curves and statistical details see Tables S1 and S3

The average costs of a collecting visit (calculated from the amount of CO_2_ release and the duration of the visit) depended also on ambient temperature (GLM statistics, Table S2) and decreased in an exponential decay with *T*_a_ (*p* < 0.0001, ANOVA). At 12 °C, in the shade, the costs of a collecting visit amounted to 10.4 J and decreased to 2 J at 30 °C. At 40 °C, when the bees collected in the sun, it amounted to only 0.5 J (Fig. 3d). The total costs of collecting depended strongly on the duration of the collecting stays in the shade (Fig. 4; *p* < 0.0001, ANOVA), and to a lesser extent, but still significantly, on the duration of the collecting stays in the sun (Fig. 4; *p* < 0.05, ANOVA). The constants of the fit curves and detailed statistics for the duration and the costs of collecting are provided in Tables S1, S2 and S3.

The crop load with water, determined exemplary in one bee, was 55.4 ± 2.75 mg (*n* = 10 foraging stays, *T*_a_ = 24.0 °C) and was comparable with results of Kovac et al. (2010), amounting to 56.6 ± 7.62 mg (*n* = 110 stays and bees).

## Discussion

In the course of our comprehensive investigation, we determined the energetic effort of honeybees at a water source. We measured simultaneously body temperature and metabolic rate (CO_2_ release as energetic equivalent) of bees collecting water at an artificial water source over a wide range of ambient temperatures (Fig. 3, *T*_a_ = 12–40 °C). These results were compared with data of sucrose foraging bees from a former study (Stabentheiner and Kovac 2016) to assess the energetic effort of the water collectors and to test the “motivation hypothesis” of foraging in bees. Note that this does not include the costs of flight and of the stay inside the colony.

The “motivation hypothesis” postulates that the readiness to forage and the invested energetic effort of the foragers depends both on the quality of a food source and the demand in the colony (e.g. Dyer and Seeley 1987; Stabentheiner and Schmaranzer 1986, 1987; Schmaranzer and Stabentheiner 1988; Stabentheiner and Hagmüller 1991; Stabentheiner et al. 1995; Balderrama et al. 1992; Moffatt and Núñez 1997; Moffatt 2000, 2001; Stabentheiner 1996, 2001; Sadler and Nieh 2011; Stabentheiner and Kovac 2014, 2016). The quality of a source is, for example, determined by the amount and concentration of nectar, the number of flowers at a patch and the distance of the patch from the hive. In detail, the body temperature of sucrose foraging honeybees at a feeding place was dependent on the concentration of the sugar solution (Stabentheiner and Schmaranzer 1986, 1987; Dyer and Seeley 1987; Schmaranzer and Stabentheiner 1988; Waddington 1990). The body temperature of foragers performing waggle or round dances to recruit other bees inside the colony depended on the sucrose concentration (Stabentheiner and Hagmüller 1991; Stabentheiner et al. 1995) and the foraging distance (Stabentheiner 1996). In addition, parameters inside the colony, e.g. the honey or pollen stock and the actual demand in the hive (Stabentheiner 2001), as well as parameters outside the colony like season or environmental conditions influence the bees’ motivational status (e.g. Kovac and Schmaranzer 1996; Germ et al. 1997; Stabentheiner 2001).

In social insects, individual foragers balance energy expenditure of their foraging trips with the net energetic gains to the colony (e.g. Seeley et al. 1986, 1991; Varjú and Núñez 1991; Balderrama et al. 1992). However, concerning energetics, water collecting is a special case, as water does not contain any energetic value. In this case, we suggest the motivation to be solely dependent on the colony’s actual need for water. Our results deliver strong evidence for this hypothesis, as the variability of the evaluated parameters (body temperature and metabolic rate, Fig. 3a, b) was very high. We conducted the experiments in spring and summer, on cold and warm days. The collected water was probably used for different purposes, for diluting stored honey or for cooling the hive. As 10 colonies were housed near the experimental area, we presume that the bees collecting at our water source originated from different colonies. The results, therefore, most probably reflect the colonies’ differing demand for water. Especially in spring, after a period of bad weather, the bees need water very urgently for providing the brood, and foragers have been observed sometimes in large numbers at water sources at very low temperatures (below 10 °C, Kovac et al. 2010; Chilcott and Seeley 2018, and personal observation). The present data show convincingly that even at low temperatures, when water was surely not used for cooling, bees exhibited a very high body temperature (mean *T*_thorax_ = 37.5 °C at *T*_a_ = 15 °C; Fig. 3b). The thorax temperature at this low ambient temperature was very similar to that of water collecting bees measured by Schmaranzer (2000) and Kovac et al. (2010), but higher than in nectar foragers (Kovac and Stabentheiner 2011). This high body temperature can be regarded as a good indicator of the bees’ high motivation.

Differences were observed in the results obtained in the two experimental seasons. The bees investigated in the first series, which were measured in spring 2009 (April), needed longer for their collecting trips than the bees investigated in the second series in summer 2010 (Fig. 3c). They exhibited somewhat lower body temperatures, especially of the head, which reduces the suction speed (Kovac et al. 2010) and thus prolongs the foraging time. In the end, however, the total costs for collection (Fig. 3d) in 2009 were similar to those in 2010 although the metabolic rate was somewhat lower (Fig. 3a). We presume a lower motivation to gather water during the spring 2009 measurements and, therefore, a physiological limitation due to the lower head temperature, to be responsible for these differences.

To assess the bees’ energetic investment, we compared our data of water collectors with data of sucrose foraging bees (by courtesy of Stabentheiner and Kovac 2016). This comparison revealed surprising results. The energetic performance of the water collectors is very similar to that of bees foraging 0.5 M sucrose solution of unlimited flow (Fig. 3). Although we could detect significant differences in the thorax temperature, which in the shade were in part even higher in water collectors (Fig. 3b, *p* < 0.0001, ANOVA), the metabolic rate was quite similar and not significantly different (Fig. 3a, *p* > 0.05, ANOVA). The duration of sucrose foraging trips was somewhat longer (Fig. 3c, *p* < 0.01, ANOVA) and therefore, the total costs of the foraging stays were higher during sucrose foraging (Fig. 3d, *p* < 0.001, ANOVA). The higher viscosity of the sucrose solution impairs the bees’ suction speed and is probably responsible for the slightly prolonged duration of foraging (Nicolson et al. 2013; Stabentheiner and Kovac 2016). The correlation between the foraging costs and the duration of a foraging stay was nearly identical in water collectors and sucrose foragers in the shade (Fig. 4). These results demonstrate that water collectors exhibit an energetic investment comparable to sucrose (or nectar) foragers, although they yield no energetic gain. Lindauer (1954) and Kühnholz and Seeley (1997) showed that a colony’s (urgent) need for water stimulates foragers to collect water and that the motivation of the foragers to continue collecting water depends on the time needed inside the hive to unload their crop content. We suggest these foragers to be strongly motivated and in further consequence to perform a high energetic investment, which results in a high body temperature. But why do they need a high body temperature? They could also collect at lower temperatures and with lower energy expenditure. However, a high body temperature improves general agility (Stabentheiner et al. 2003), enables fast foraging and higher loads to be carried due to improved flight muscle function (Coelho 1991), and the bees’ suction speed directly depends on a high head temperature (shown in water collecting bees by Kovac et al. 2010). Fast drinking reduces the duration of the collecting stay and increases the total intake rate (foraging trips per time). If the colonies’ demand for water decreases, the time for the foragers to unload their crop content increases (Lindauer 1954; Kühnholz and Seeley 1997) and their motivation will decrease simultaneously. Subsequently they will reduce their energetic effort and in the end stop foraging.

We could show that the energetic investment of foraging bees not only depends on the gain of energy or protein but that it is modulated by the colonies’ demand for a resource in general, even a special one like water.

## Electronic supplementary material

Below is the link to the electronic supplementary material.

Supplementary material 1 (PDF 59 KB)
