# Supplementary material for: The energetics and thermoregulation of water collecting honeybees
Source: J Comp Physiol A Neuroethol Sens Neural Behav Physiol. 2018 Aug 6;204(9):783–90. doi: 10.1007/s00359-018-1278-9 (PMC6182700; doi:10.1007/s00359-018-1278-9)
Supplement: Supplementary file 1 — Supplementary material 1 (PDF 59 KB) [file 359_2018_1278_MOESM1_ESM.pdf]

Journal of Comparative Physiology A

The energetics and thermoregulation of water collecting honeybees

Helmut Kovac, Helmut Käfer, Anton Stabentheiner
Institute of Biology, University of Graz, Universitätsplatz 2, A-8010 Graz, Austria

E-Mail: helmut.kovac@uni-graz.at, anton.stabentheiner@uni-graz.at

Supplementary Table S1. Constants and statistics for regression functions in Figs 3 and 4.

Figure	Radiation (Wm^{-2})	Function	Parameters					N	R^2	P
		linear	A	B						
		exponential	A	B						
		polynomial	A	B	C					
		cubic	A	B	C	D				
		exp. decay	y0	A1	t1	k	tau			
Fig. 3a	<350	polynomial	-3.264	17.58329	-0.44261			183	0.05748	0.0018
	>350	linear	283.55704	-5.57613				21	0.17163	0.03518
Fig. 3b	<350	polynomial	23.19673	1.36806	-0.02716			190	0.18194	<0.0001
	>350	polynomial	102.66239	-3.92493	0.06049			40	0.27380	0.00102
Fig. 3c	<350	exp. decay	37.38638	1008.82337	6.15496	0.16247	4.26629	190	0.66445	<0.0001
	>350	linear	66.02438	-1.01283				40	0.24017	<0.0001
Fig. 3d	<350	exponential	1269.24033	0.91594				183	0.59445	<0.0001
	>350	linear	212.80273	-4.78674				21	0.42523	<0.0001
Fig. 4	<350	linear	-0.16718	0.05903				183	0.71097	<0.0001
	>350	linear	-0.25154	0.04182				21	0.18663	0.02888

Definition of functions: Linear, $y=A + B*x$. Exponential, $y=A*B^x$, Polynomial, $y=A + B*x + C*x^2$, Cubic, $y=A + B*x + C*x^2 + D*x^3$. Exponential (exp.) decay, $y=y0 + A1*e^{(-x/t1)}$; derived parameters: decay rate, k=1/t1; half life, tau=t1*ln(2). N=number of stays (visits). ANOVA, df=N-2 for linear and exponential, N-3 for exponential decay and polynomial, N-4 for cubic functions.

Supplementary Table S2. Summary of General Linear Model statistics for all measured and calculated parameters in Figs 3a-d.

**Metabolic rate (Fig. 3a)**	Sum of squares	DF	Mean square	F-Quotient	P-Value
Model	55.5451	2	27.7725	48.19	<0.0001
Residuals	145.819	253	0.576358		
Total (corrected)	201.364	255			
Covariable					
Temperature	0.653562	1	0.653562	1.13	0.2879
Radiation	42.4028	1	42.4028	73.57	<0.0001
Residuals	145.819	253	0.576358		
Total (corrected)	201.364	255			
**T_{thorax} (Fig. 3b)**					
Model	291.606	2	145.803	37.67	<0.0001
Residuals	1246.34	322	3.87062		
Total (corrected)	1537.95	324			
Covariable					
Temperature	139.648	1	139.648	36.08	<0.0001
Radiation	6.48043	1	6.48043	1.67	0.1966
Residuals	1246.34	322	3.87062		
Total (corrected)	1537.95	324			
**Duration (Fig. 3c)**					
Model	310082.0	2	155091.0	141.44	<0.0001
Residuals	413490.0	377	1096.53		
Total (corrected)	723572.0	379			
Covariable					
Temperature	184697.0	1	184697.0	168.44	<0.0001
Radiation	100.216	1	100.216	0.09	0.7626
Residuals	413390.0	377	1096.53		
Total (corrected)	723572,0	379			
**Costs (Fig. 3d)**					
Model	1.61559E6	2	807796.0	147.67	<0.0001
Residuals	1.38399E6	253	5470.34		
Total (corrected)	2.99959E6	255			
Covariable					
Temperature	1.01443E6	1	1.01443E6	185.44	<0.0001
Radiation	2504.6	1	2504.6	0.46	0.4992
Residuals	1.38399E6	253	5470.34		
Total (corrected)	2.99959E6	255			

Supplementary Table S3. Summary of ANOVA statistics for comparison of water collecting bees with sucrose foraging bees (0.5 M in shade, from the study of Stabentheiner and Kovac 2016) of the measured and calculated parameters in Figs 3 and 4 (y-axes) in dependence on ambient temperature (T_a, x-axes) and duration.

Figure	Parameter	Effect	Quadrate sum	df	X^2	F-Quotient	P-Value
Fig. 3a	Metabolic rate	T_a	59798.6	1	59798.6	43.58	<0.0001
		Intercept	1661.93	1	1661.93	1.21	0.2718
		Slope	431.206	1	431.206	0.31	0.5754
		Model	61891.7	3			
Fig. 3b	T_{thorax}	T_a	225.748	1	225.748	71.79	<0.0001
		Intercept	120.428	1	120.428	38.3	<0.0001
		Slope	0.965872	1	0.965872	0.31	0.5797
		Model	347.142	3			
Fig. 3c	Duration	T_a	251734	1	251734	830.21	<0.0001
		Intercept	2516.86	1	2516.86	8.3	0.0042
		Slope	974.415	1	974.415	3.21	0.0738
		Model	255225	3			
Fig. 3d	Costs	T_a	2455640	1	2455640	973.94	<0.0001
		Intercept	29765.8	1	29765.8	11.81	0.0007
		Slope	14341.4	1	14341.4	5.69	0.0176
		Model	2499750	3			
Fig. 4	Costs	Duration	945.887	1	945.887	1241.24	<0.0001
		Intercept	0.0717916	1	0.0717916	0.09	0.7591
		Slope	0.0221817	1	0.0221817	0.03	0.8646
		Model	945.981	3			
